# Enhancement of Congo Red-Neomycin Resonance Rayleigh Scattering by Dodecyl Trimethyl Ammonium Bromide and its Application

**DOI:** 10.1155/2022/6970747

**Published:** 2022-08-27

**Authors:** Fanfan Zhang, Yangyang Chen, Dan Zhang, Yang Jia, Junsheng Meng, Lirong Jiang, Shengke Yang

**Affiliations:** ^1^Key Laboratory of Subsurface Hydrology and Ecology in Arid Areas, Ministry of Education, Chang'an University, Xi'an 710054, China; ^2^School of Water and Environment, Chang'an University, Xi'an 710054, China; ^3^China Jikan Research Institute of Engineering Investigations and Design Co, LTD, Xi'an 710000, China

## Abstract

A simple, rapid, and convenient method for the determination of neomycin based on the ion association method was proposed. In Britton–Robinson buffer solution, neomycin could react with Congo red to form an ionic association, which in turn reacted with dodecyl trimethyl ammonium bromide to form a ternary ionic association. The three were combined in a 1 : 1 : 1 ratio, which significantly enhanced the resonant Rayleigh scattering intensity at 468 nm. The obtained resonant Rayleigh scattering sensor showed a linear relationship with neomycin in the range of 0.07∼1 *μ*g·mL^−1^. The limit of detection was 0.02 *μ*g·mL^−1^, and the limit of quantification was 0.037 *μ*g·mL^−1^. The experimental conditions were optimized. The method was verified based on the ICH rule. The established method could be applied to the analysis of the acceptable recovery rate of neomycin in powdered veterinary drugs.

## 1. Introduction

Neomycin (NEO) is a water-soluble aminoglycoside antibiotic produced by the fermentation of actinomycete streptomycin [1, 2]. It has a bactericidal effect on Gram-negative bacteria and some Gram-positive bacteria [3]. Neomycin is commonly used to treat bacterial keratitis, conjunctivitis, blepharitis, and otitis media [4]. It can accumulate in animal tissues, which may cause allergic reactions, tissue toxicity, intestinal flora disorders, and antibiotic resistance. It can also cause damage to the human kidneys and cranial nerves [5]. Therefore, the massive use of neomycin will inevitably pose a threat to human health. The determination methods of neomycin include liquid chromatography [6, 7], high-performance liquid chromatography [8], electrochemical analysis [9], immunochromatographic assay [10], enzyme-linked immunosorbent assay [11], fluorescence method [12, 13], and chemiluminescence method [14]. However, some of these methods are time-consuming, some could pollute the environment, some are expensive, and so on. Therefore, it is of great significance to establish a simple and sensitive detection method for neomycin.

Resonance Rayleigh scattering (RRS) is a special type of elastic scattering with special synchronous fluorescence when the wavelength of the Rayleigh scattering band is at or close to the molecular absorption band [15, 16]. RRS has been widely used in the determination of heavy metals [17], organic substances [18], drugs [19, 20], etc., and has been applied in the detection of neomycin.

After years of development, RRS has been widely used in the determination of trace substances [18–20], and has been applied to the study of neomycin. Liu et al. found that thioglycolic acid-encapsulated cadmium sulfide quantum dots can form large aggregates with neomycin sulfate under the action of electrostatic attraction and hydrophobic force, thereby significantly enhancing resonant Rayleigh scattering [21]. Qiao et al. used various aminoglycoside antibiotics including neomycin to interact with calf thymus DNA and established a method for detecting calf thymus DNA by resonance Rayleigh scattering [22]. Ouyang et al. established a resonance Rayleigh scattering method for the detection of neomycin sulfate using copper oxide nanosol as a probe [4]. In summary, RRS is a sensitive, cheap, fast, convenient, and easy-to-operate detection method [23]. Based on the advantages of the RRS method and certain preliminary explorations, this research established an RRS method for the detection of neomycin.

Congo red (CR) is a typical direct azo dye of benzidine, which is widely used in medical tests as a biological stain [24]. CR exists as an anion in water. Due to the hydrophobic interaction between the aromatic rings of the dye, it has a tendency to aggregate and form supramolecular systems (ribbon micelles) of different sizes and shapes [25]. It can interact with cationic surfactants such as cetyltrimethylammonium bromide and cetyl pyridinium bromide [26], and can also form complexes with amyloid [27, 28]. Congo red, as a targeted substance, has great potential in combination with aminoglycoside antibiotics.

Surfactants in a large number of washing products will eventually enter the water environment. Due to their hydrophilic and hydrophobic functions, they can change the surface properties of other substances. In the research related to RRS, Gao et al. established a method for the determination of bovine serum albumin by using the difference between the binding of cationic surfactant cetyl trimethyl ammonium bromide and anionic surfactant sodium dodecyl benzene sulfonate to bovine serum albumin [29]. Sun et al. found that non-ionic surfactants have little effect on the RRS strength of chitosan and Acid Blue 119 [30]. Zou et al. found that cationic surfactants could weaken the intensity of chitosan-signature Eosin Y resonance Rayleigh scattering, while anionic surfactants and nonionic surfactants could enhance the RRS of the system [31]. It can be seen that different types of surfactants would participate in the association reaction with their unique properties, change the surface hydrophilic and hydrophobic properties of the association, and significantly affect the RRS strength. Different types of surfactants have different influence mechanisms in different systems. With the aid of resonance Rayleigh scattering, it is highly sensitive and feasible to carry out the combined characteristics of different types of surfactants and a variety of pollution.

In this work, CR and NEO formed an ionic association in a 1 : 1 ratio under electrostatic attraction and hydrophobic interaction, resulting in a significant enhancement of the RRS intensity at 469 nm. After adding dodecyl trimethyl ammonium bromide (DTAB), the three would be associated in a ratio of 1 : 1 : 1, which further enhanced the RRS strength of the system. The NEO concentration was in a linear relationship with NEO concentration in the range of 0.07∼1 *μ*g·mL^−1^. A spectroscopic method with high sensitivity, good selectivity, and rapid determination of NEO was established. The generation mechanism of the RRS signal was studied. The method was applied to the determination of NEO in veterinary drugs with satisfactory results.

## 2. Materials and Methods

### 2.1. Apparatus

An F97Pro fluorescence spectrophotometer (Prism, Shanghai, China) equipped with a xenon lamp was used to obtain RRS. Absorption spectra were recorded by a UV-2450 UV-Vis spectrophotometer (Tsushima, Japan). The Malvern Laser Particle Size Analyzer ZEN3700 (UK) was used for charge measurements.

### 2.2. Reagents

Phosphoric acid (H_3_PO_4_), boric acid (H_3_BO_3_), acetic acid (CH_3_COOH), sodium hydroxide (NaOH), and hydrochloric acid (NaCl) were obtained from Sinopharm Chemical Reagent Company (Beijing, China). Neomycin, Congo red, and dodecyl trimethyl ammonium bromide were obtained from Aladdin (Shanghai, China). Tetradecyl trimethyl ammonium bromide (TTAB), acetyl trimethyl ammonium bromide (CTAMB), polyethylene glycol 400 (PEG400), polyethylene glycol 6000 (PEG6000), dodecyl sodium benzene sulfonate (SDBS) and sodium dodecyl sulfonate (SDS) were purchased from Shanghai Macklin Biochemical Company. The veterinary drug (neomycin sulfate soluble powder) was purchased from QianFang Animal Health Technology Co., Ltd., China market.

The experimental water was ultrapure water produced by Milli-Q ultrapure water system (Millipore, USA).

### 2.3. Characterization of Neomycin

H_3_PO_4_, H_3_BO_3,_ and CH_3_COOH at a concentration of 0.04 mol·L^−1^ were mixed with NaOH (0.2 mol·L^−1^) to form a Britton–Robinson buffer solution (BR buffer solution) at pH 6. Add 1 mL of pH 6.0 BR buffer solution, 1 mL of 1.5 × 10^−4^ mol·L^−1^ CR, an appropriate amount of 10 *μ*g·mL^−1^ NEO, and 1 mL of 1.0 × 10^−4^ mol·L^−1^ DTAB to a 10.0 mL calibration flask, then titrate to the mark with water.

**Figure 1:**
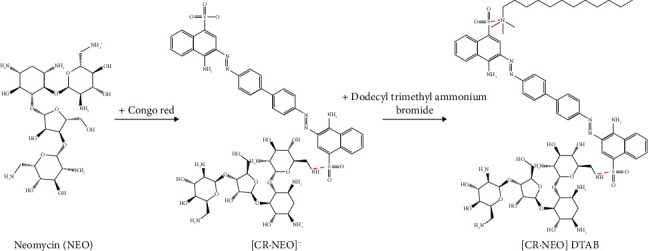
The reaction process of neomycin detection.

Then, a simultaneous scan in the wavelength range from 325 to 600 nm was performed on a spectrofluorometer to obtain resonance scattering spectra. The detector voltage and slit width were set to 600 V and 10 nm, respectively. The reaction process is shown in Figure 1. The signal intensity at 469 nm is *I*, and the blank solution without neomycin is *I*_0_. The varying resonance scattering intensity Δ*I* = *I* − *I*_0_ was calculated. These results are reported as the mean ± standard deviation based on 5 measurements.

### 2.4. Procedures for the Estimation of NEO in Veterinary Drugs

The method for the determination of NEO content in veterinary drugs: Weigh 20 mg sample in a bag of powder veterinary medicine and quantitatively add it into a 100 ml calibration bottle. Disperse the sample in about 30 ml of ultrapure water and sonicate for 15 minutes at room temperature. Dilute the solution with ultrapure water to the desired concentration. The NEO concentration was detected by the resonance Rayleigh scattering method, which was repeated five times.

## 3. Results and Discussion

### 3.1. The Resonant Rayleigh Scattering Spectra

**Figure 2:**
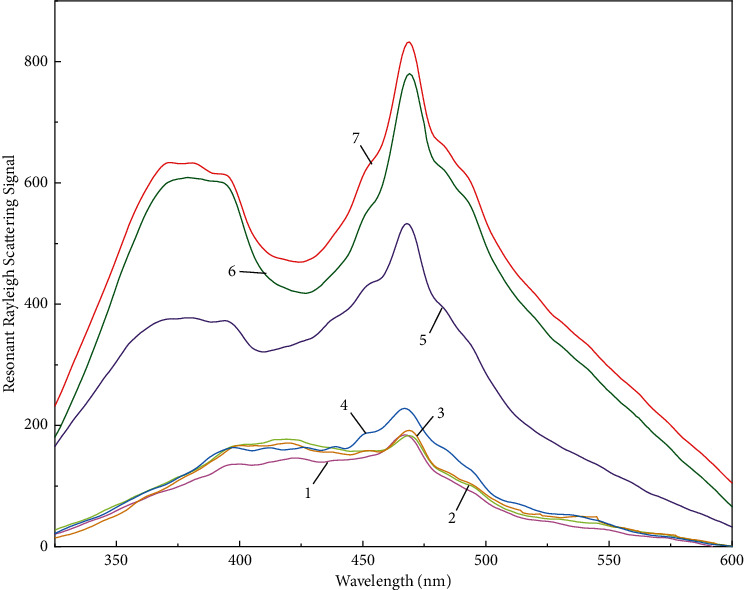
Resonant Rayleigh scattering spectra of different systems. 1 mL pH 6.0 Britton-Robinson buffer solution, 1: DTAB; 2: congo red; 3: neomycin 4: neomycin-DTAB; 5: congo red-DTAB; 6: congo red- neomycin; and 7: congo red-neomycin-DTAB. The concentrations of the added substances were 1.0 × 10^−5^ mol·L^−1^ for DTAB, 1.5 × 10^−5^ mol·L^−1^ for congo red, 1.0 *μ*g·mL^−1^ for neomycin.

**Figure 3:**
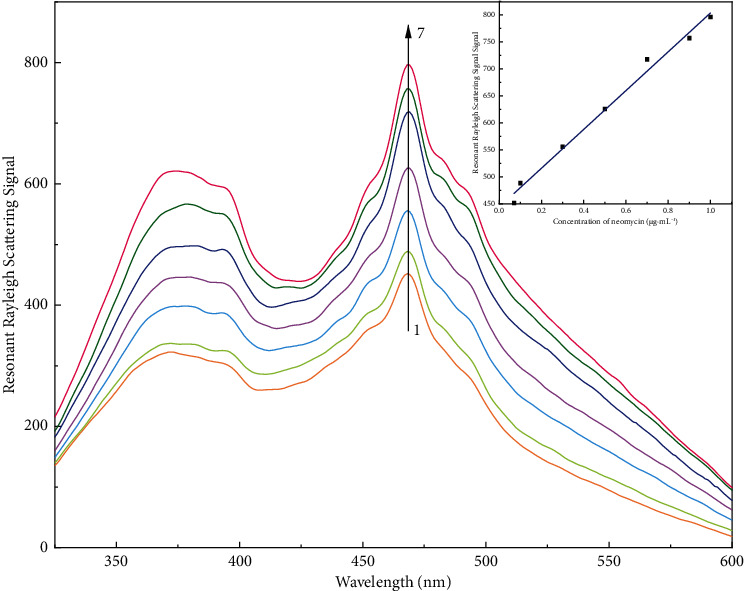
Resonance Rayleigh scattering spectra of congo red-neomycin-DTAB system. 1–7: 1 mL pH 6.0 Britton–Robinson buffer solution, congo red: 1.5 × 10^−5^ mol·L^−1^, neomycin: 0.07, 0.1, 0.3, 0.5, 0.7, 0.9, 1.0 *μ*g·mL^−1^, DTAB: 1.0 × 10^−5^ mol·L^−1^. The inset shows the linear range of neomycin.

Figure 2 shows the resonant Rayleigh scattering spectrum of different systems. It could be seen that the RRS intensities of CR, NEO, and DTAB were very weak. However, the RRS intensity of the binary compound [CR·NEO]^−^ was high. The RRS would be further enhanced after the binary compound reacted with DTAB to form a ternary ion-associative complex. The maximum RRS peak was located at *λ* = 468 nm. The enhancement of the RRS intensity of the [CR·NEO] DTAB system was proportional to the concentration of NEO (Figure 3). Therefore, a new NEO assay method was established.

### 3.2. Optimal Reaction Conditions

#### 3.2.1. Effect of pH and Dosage of Buffer Solution

It is known that CR and NEO exist in an ionic state under acidic conditions. At this time, they are more likely to form a ternary ion association with the cationic surfactant DTAB. Therefore, we used a BR buffer solution to test the effect of pH. The results are shown in Figure S1. As the pH of the added buffer solution increased, the ∆*I* value of the system gradually increased, which reached the highest at pH 6.0, and ∆*I* decreased at pH 7.0. The Zeta potential of the solution system was measured under the conditions of pH 2.0, pH 5.0 and pH 6.0, and the results were −5.32 mV, −2.13 mV and −2.04 mV, respectively. It is well known that the lower the Zeta potential (absolute value) is, the easier it is for the related constituents to agglomerate or aggregate. That is, the attractive force exceeds the repulsive force [30]. At pH 5.0, the Zeta potential was very close to that at pH 6.0, which meant that there was no significant difference in the binding attraction between CR, NEO, and DTAB at these two pH values. When the pH value was 2.0, the effective combination of CR, NEO, and DTAB was lower, resulting in a larger absolute potential value. In addition, the effect of different dosages of pH 6.0 BR buffer solution was tested (Figure S2), and it was found that the ∆*I* was the highest when the dosage was 1 ml. Therefore, 1 ml of BR buffer solution with pH 6.0 was added for subsequent experiments.

#### 3.2.2. Effect of Temperature

The influence of heating temperature on the interaction system was discussed. It could be seen in Figure S3 that *I*_0_ and *I* of the system remained stable with the increase of temperature in the range of 15–35°C, so the change of ∆*I* was also very small. The absolute value of the potential varies very little over this temperature range. That said, there was little change in attractiveness between CR, NEO, and DTAB. Therefore, we chose room temperature as the subsequent experiment temperature.

#### 3.2.3. Effect of Congo Red Concentration

The results of the CR concentration experiment (Figure S4) showed that with the increase of the added CR concentration, the *I* of the system first increased rapidly, and then the rate of increase slowed down, while the rate of increase of *I*_0_ was slow. Therefore, ∆*I* first increased and reached a high value when the CR concentration was 1 × 10^−5^ mol·L^−1^, and then changed insignificantly. This was because when the CR concentration was less than 1 × 10^−5^ mol·L^−1^, the interaction was not complete due to the lack of CR. When CR was added in excess, the interaction was complete, and the remaining CR might undergo slight self-aggregation, so the change in ∆*I* was not significant. The concentration of CR added in subsequent experiments was 1.5 × 10^−5^ mol·L^−1^.

#### 3.2.4. Effect of Dodecyl Trimethyl Ammonium Bromide Concentration

The results of the DTAB concentration experiment (Figure S5) showed that as the concentration of added DTAB increased, the *I* increase the rate of the system was fast while the *I*_0_ increase rate was slow. Therefore, ∆*I* also increased with increasing DTAB concentration. It was observed that when the DTAB concentration was 1 × 10^−5^ mol·L^−1^, the ∆*I* of the system reached a higher value, and the interaction between DTAB and the system was relatively complete. Therefore, 1 × 10^−5^ mol·L^−1^ was selected as the concentration of DTAB for subsequent experiments.

#### 3.2.5. Reaction Time and Stability

The stabilization time under optimal conditions was studied (Figure S6). The results show that the reaction time was 30 minutes, and the intensity change was less than 5% within 2 hours. Therefore, the follow-up experiment selected the reaction time as 30 minutes.

#### 3.2.6. Effect of Add Sequence

Considering that both CR and NEO are dissociated in acidic medium and exist as ions. Under the optimal reaction conditions, after adding 1 ml of pH 6.0 BR buffer solution, the effect of different addition order (Table S1) of CR, NEO, CTAB on the strength of RRS was studied (Figure S7). It was found that the order of addition had little effect on the ∆*I* of the system, and the order of addition of CR-NEO-DTAB had the strongest RRS.

#### 3.2.7. Effect of Ionic Strength (Calculated as NaCl)

The influence of ionic strength (calculated as NaCl) on the RRS strength of the system was studied (Figure S8). It could be seen that as the concentration of added NaCl increased the RRS intensity of the system decreased. This indicated that CR, NEO, and DTAB were combined mainly by electrostatic attraction. Too high ionic strength would interfere with the combination of the three. Therefore, the ionic strength of the solution must be strictly controlled during the measurement process.

#### 3.2.8. Effect of Different Surfactants on Resonant Rayleigh Scattering Strength of Congo Red-Neomycin System

In this experiment, the effects of cationic surfactants, nonionic surfactants, and anionic surfactants on the “CR - NEO” system were investigated.

**Figure 4:**
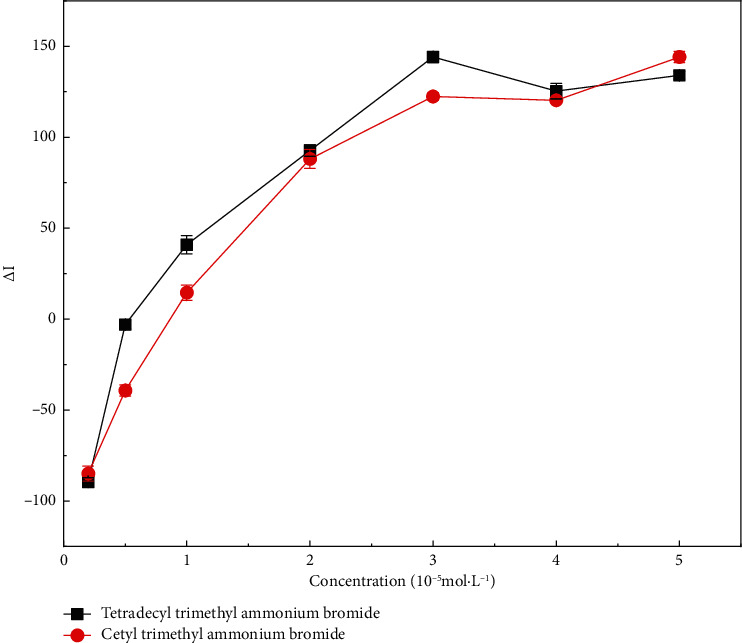
Resonant Rayleigh scattering spectra after addition of cationic surfactant to congo red-neomycin system. The concentration of tetradecyl trimethyl ammonium bromide and cetyl trimethyl ammonium bromide: 0.2, 0.5, 1, 2, 3, 4, 5 × 10^−5^ mol·L^−1^.

Figure 4 showed that as the concentration of the added cationic surfactant increased, the RRS strength of the resulting ternary system increased. This indicated that cationic surfactants could form ion associations with CR-NEO through electrostatic interaction, which increased the molecular volume and enhanced RRS. And the effect of TTAB in enhancing RRS was better than CTAMB. This was because the larger molecular weight of CTAMB lead to its obvious steric hindrance effect. Therefore, CTAMB was not easy to react with the original system, and the enhancement effect on the RRS of the system was relatively weak.

**Figure 5:**
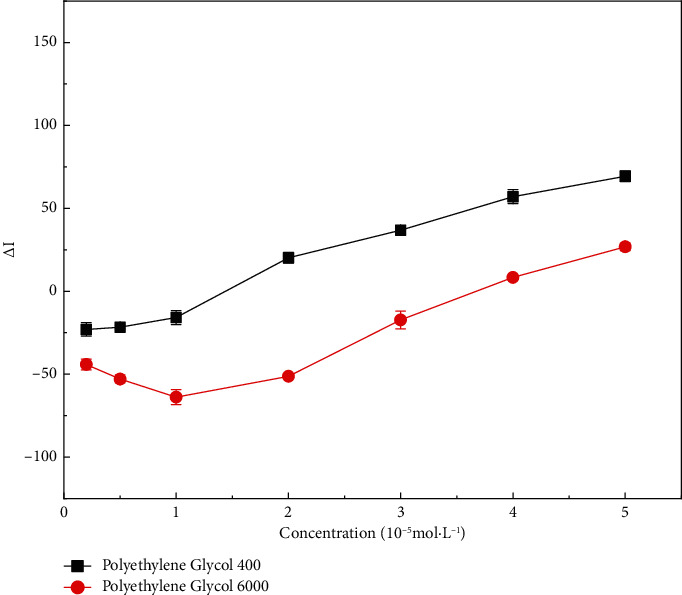
Resonant Rayleigh scattering spectra after addition of nonionic surfactant to congo red-neomycin system. The concentration of polyethylene glycol 400 and polyethylene glycol 6000 : 0.2, 0.5, 1, 2, 3, 4, 5 × 10^−5^ mol·L^−1^.

The RRS intensity of the ternary system increased with increasing polyethylene glycol concentration (Figure 5). Polyethylene glycol is an electrically neutral substance that has a dispersive effect, causing itself to be positively charged. Adding positively charged substances to the CR-NEO system would neutralize the original charge and reduce its hydrophilicity. Therefore, polyethylene glycol could increase the resonant Rayleigh scattering intensity of the system.

**Figure 6:**
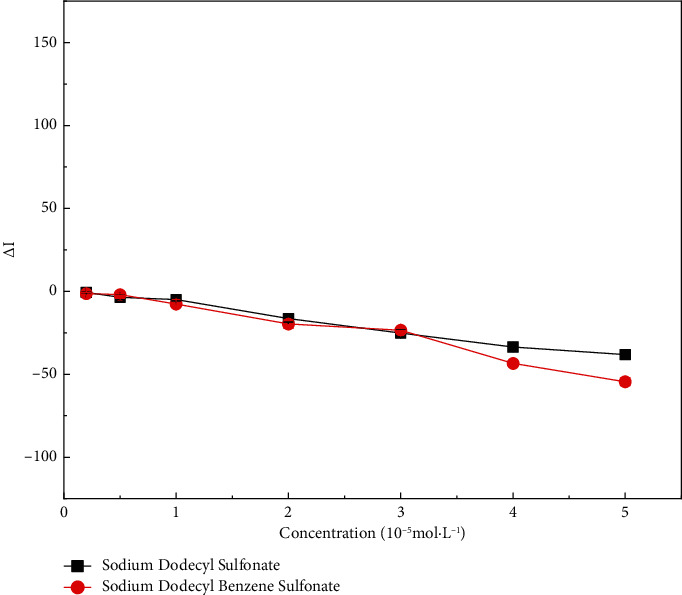
Resonant Rayleigh scattering spectra after addition of anionic surfactant to congo red-neomycin system. The concentration of sodium dodecyl benzene sulfonate and sodium dodecyl sulfonate: 0.2, 0.5, 1, 2, 3, 4, 5 × 10^−5^ mol·L^−1^.

The effects of sodium dodecyl benzene sulfonate and sodium dodecyl sulfonate on the RRS of the CR-NEO system were explored (Figure 6). We found that they had less effect on the RRS of the system. This indicated that the anionic surfactant would not interact with the CR-NEO system.

### 3.3. Mechanism of Surfactant Action on Congo Red-Neomycin System

The resonance Rayleigh scattering formula was derived from the formula that the stability constant of the complex and the resonance light intensity was proportional to the concentration of scattered ions [32, 33]:

(1) $$\begin{array}{c} \frac{I}{I_{a}}-1=K_{A}\left[P\right],\\ \log\left[\frac{\left(I-I_{a}\right)}{I_{a}}\right]=m\;\log\left[P\right]+\log\;K_{A}, \end{array}$$

where *I*_*a*_ is the incident light intensity, *I* is the resonance Rayleigh scattering light intensity, *P* is the NEO concentration, *K*_*A*_ is the resonance binding constant, and *m* is the number of binding sites. Thus, the combination ratio of CR and NEO was 1 : 1, the combination ratio of CR, NEO, and DTAB was 1 : 1 : 1, and the composition of the ternary ion association was [CR·NEO] DTAB. The reaction mechanism of the complex was as follows:

(2) $$\begin{array}{c} {\text{CR}2}^{-}+{\text{NEO}}^{+}⇄{\left[\text{CR}\cdot \text{NEO}\right]}^{-},\\ {\left[\text{CR}\cdot \text{NEO}\right]}^{-}+{\text{DTAB}}^{+}⇄\left[\text{CR}\cdot \text{NEO}\right]\text{DTA.} \end{array}$$

Congo Red dissociates the sodium ion on the sulfonamide group in the acidic aqueous solution, so it exists as an anion. Neomycin is formed by the condensation of amino cyclic alcohol and amino sugar, and exists in the form of cations in acidic aqueous solutions. Under the action of electrostatic attraction, the two combined in a ratio of 1 : 1 to form an ion association. After the two were combined, they were negatively charged, so they could further combine with the cationic surfactant DTAB to form a ternary ion association.

**Figure 7:**
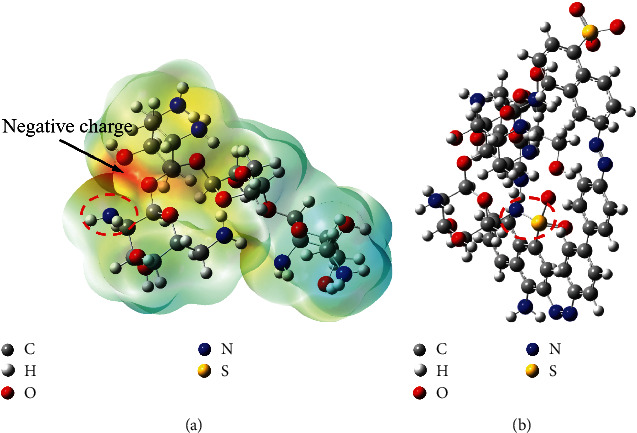
Neomycin electrostatic potential distribution and structure diagram of Congo red-neomycin system. (a) The orbital charge distribution and electrostatic potential of the natural bond of neomycin. (b) The structural diagram of the congo red-neomycin system.

The Gaussian 09 program [34] was used for optimization and frequency calculations to verify the stable structure of the neomycin molecule. Then, based on the optimized geometry, natural bond orbital and electrostatic potential analyses were performed (Figure 7). Different surface electrostatic potential values are represented by different colors: red, blue, and green represent the regions with the most negative, most positive, and zero electrostatic potential, respectively. From the electrostatic potential map of neomycin, it can be seen that the most easily combined N atom site with Congo red. And the structure of the Congo red-neomycin system was optimized.

In addition, we got the combination ratio of CR, NEO, and non-ionic surfactant FEG400 of 1 : 1 : 0.5. PEG400 might be combined with the CR-NEO system through adsorption. The resonance binding equation of the anionic surfactant SDBS and CR-NEO system is: *y* = −0.0028 *x* + 1. It indicated that SDBS would not interact with the original system, and no new complexes were formed.

### 3.4. Resonance Rayleigh Scattering Enhancement Mechanism

#### 3.4.1. Spectral Analysis of the System

As shown in Figure 2, the scattered light intensity of individual CR, NEO, and DTAB were very low. The RRS intensity of the NEO-DTAB system was still low. This indicated that although the two could be adsorbed by hydrophobic force, the binary complex formed was still linear in the solution, so the RRS value of the solution change a little significantly. The resonance Rayleigh scattered light intensity of CR-DTAB and CR-NEO was high. The intensity of resonance Rayleigh scattered light of CR-NEO-DTAB was the highest. This was because CR existed in the form of anions in acidic solutions, while NEO existed in the form of cations, and the two could form tight ion associations under the action of electrostatic attraction. CR could also interact with cationic surfactants to form mixed aggregates. The three could form a ternary ion association, further increasing the molecular volume and increasing the strength of RSS.

Zeta potential measurement results also confirmed the above conclusions. Among them, the Zeta potential of CR-DTAB was −3.04 mV, CR-NEO was −2.15 mV, and CR-NEO-DTAB was −2.04 mV. By comparing the zeta potentials of the above three cases, it could be concluded that after adding DTAB to the CR-NEO ion binding system, the volume of the complex was further increased and the system was more tightly bound.

#### 3.4.2. Absorption Band

**Figure 8:**
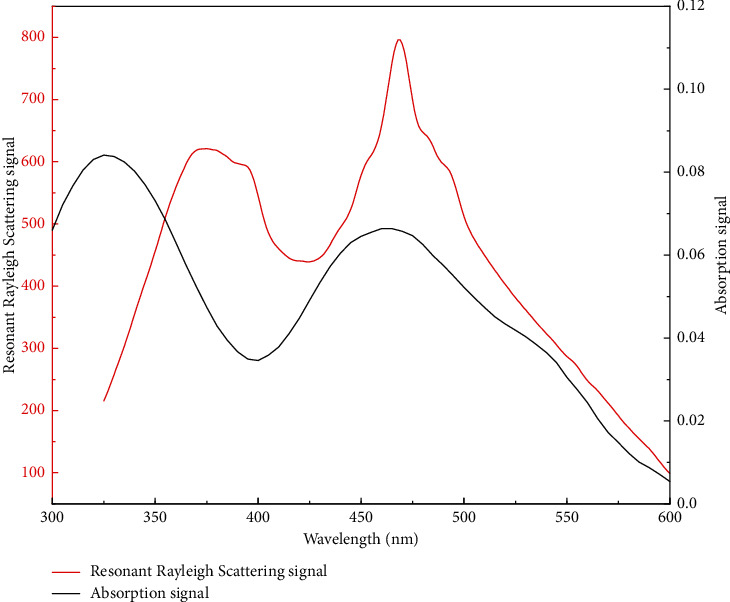
Resonant Rayleigh scattering spectrum and absorption spectrum of congo red-neomycin-DTAB. 1 mL pH 6.0 Britton–Robinson buffer solution, congo red: 1.5 × 10^−5^ mol·L^−1^; neomycin: 1.0 *μ*g·mL^−1^; DTAB: 1.0 × 10^−5^ mol·L^−1^.

When the RRS peak is located in the molecular absorption band or close to the molecular absorption band, the scattering process can absorb light energy through resonance, resulting in a heavy scattering process, and the RRS intensity is significantly enhanced. Therefore, the resonance enhancement effect is an important reason for the enhancement of the RRS intensity of the system. By comparing the absorption spectrum with the RRS spectrum (Figure 8), it was found that the RRS peaks at 325 nm and 470 nm correspond closely to the absorption peaks at 375 nm and 469 nm, respectively. Therefore, the intensity of RRS was significantly increased.

#### 3.4.3. Influence of Hydrophobic Surface Formation

Neomycin exists in the form of cations in the acidic aqueous solution, containing -NH_3_^+^, while Congo red exists in the form of CR^2−^. After the two were combined in a ratio of 1 : 1, they were negatively charged. After positively charged cationic surfactants were added to the CR-NEO system, they would neutralize their charge and lose their hydrophilicity. The formed ion association would form a hydrophobic interface with the water phase, which was beneficial to enhancing the strength of RRS.

#### 3.4.4. The Influence of Molecular Planarity and Rigidity

CR has many benzene rings. CR binds with NEO and DTAB through charge interaction, which limits the rotation of the aromatic group in the CR molecule, thereby increasing the planarity and rigidity of the molecule. The enhancement of molecular planarity and rigidity is conducive to the enhancement of RRS.

#### 3.4.5. The Effect of Molecular Volume

The increase of scattering molecule volume is beneficial to increasing scattering intensity. The molecular volume is difficult to estimate. According to the simplified Rayleigh formula,

(3) $$\begin{array}{c} I=KcMI_{0}, \end{array}$$

where *I* is the RRS intensity, *K* is a coefficient, *M* is the molecular weight, *I*_0_ is the reagent blank intensity [35]. It can be known that when other factors remain unchanged, the scattering intensity is proportional to the molecular weight of the scattering material. When the binary compound [CR·NEO]^−^ reacts with DTAB^+^ to form the ternary ion compound [CR·NEO] DTAB, the molecular weight increases from 1265.32 to 1493.76. At this time, the volume of the system increased, resulting in an increase in the resonance Rayleigh scattering intensity.

### 3.5. Methods Validation

The developed analytical methodology was assessed in line with ICH guiding principles [36]. The study included examining the linear range, accuracy, precision, method robustness, the limit of detection (LOD), and the limit of quantification (LOQ).

### 3.6. Linearity, Ranges, and Analytical Parameters

The calibration curve of the resonance Rayleigh scattering method was established based on the relationship between neomycin concentration and RRS value. The linear regression equation was obtained as *y* = 359.16 *x* + 444.27, the correlation coefficient was 0.9904, and the detection range was 0.07∼1 *μ*g·mL^−1^. The data were analyzed by least squares curve fitting. To estimate the values of LOD and LOQ, the following two formulas were used [20, 37]:

(4) $$\begin{array}{c} \text{LOD}=3.3\frac{Sa}{b},\\ \text{LOQ}=10\frac{Sa}{b}, \end{array}$$

where *b* is the slope of the calibration curve and *Sa* is the standard deviation of the intercept. The limit of detection by resonance Rayleigh scattering was 0.020 *µ*g·mL^−1^ and the limit of quantification was 0.061 µg·mL^−1^.

### 3.7. Selectivity

Based on the above RRS experimental conditions, the influence of 20 coexisting substances on the “CR-NEO-DTAB” system was investigated (Table 1). When the relative error was about 5%, the maximum allowable concentration of coexisting substances was calculated. The results show that the interference of common amino acids and some sugars is small, and the allowable amount is large. However, metal ions have a greater impact on the results, and the allowable amount is relatively small. Therefore, in the measurement of actual samples, the ionic interference was eliminated by the pretreatment method of dialysis.

### 3.8. Detection

To evaluate the utility of the RRS method in water samples, neomycin was added to the river water samples. The test was performed with the proposed method after dialysis treatment through a dialysis bag (500 MWCO). The data in Table 2 demonstrated that the added value was not significantly different from the measured value, and the mean standard deviation value of the measured value was in the range of 1.36∼3.27. This low standard deviation value confirmed the high accuracy of this method for the selective detection of neomycin in river water samples.

### 3.9. Robustness

The robustness of the suggested spectroscopic methods was evidenced by the investigation of the influence of minor changes such as pH change (6.0 ± 0.1) and changes in the volume of DTAB solution (1.0 ± 0.1 ml) for this approach. It was found that these minor changes did not produce any significant effect on the resonance Rayleigh scattering signal which proves the robustness of the suggested methodologies.

### 3.10. Application to Pharmaceutical Drug

Powdered veterinary drugs containing NEO (QianFang Animal Protection Technology Co., Ltd.) were determined by the RRS method. The measured recovery was 99.8. The drug was also assayed by another reported method [12], and the recovery obtained was statistically compared with the method presented in this study. The values of the student's *t*-tests and variance ratio *F*-tests were estimated and compared with the theoretical values at the 95% confidence interval. It could be observed that the estimates for both parameters were lower than the values listed in the table. This was considered to be a sign of the absence of any variation in the accuracy and precision between the reported method and the developed method in the detection of NEO. Moreover, the interval hypothesis was applied to compare the results of the developed and reference methods. The 95% confidence interval estimates of the *t*-test and *F*-test did not exceed the estimates listed in the table, which demonstrated that the tested method had sufficient accuracy and precision [38].

(5) $$\begin{array}{c} \theta^{2}\left[x_{1}^{2}-\frac{S_{p}^{2}t^{2}}{n_{1}}\right]-2\theta x_{1}x_{2}+\left[x_{1}^{2}-\frac{S_{p}^{2}t^{2}}{n_{2}}\right]=0. \end{array}$$

The following two equations were applied to estimate the lower limit (*θ*_*L*_) and upper limit (*θ*_*U*_):

(6) $$\begin{array}{c} \theta_{U}=\frac{-b+\sqrt{b^{2}-4ac}}{2a},\\ \theta_{L}=\frac{-b-\sqrt{b^{2}-4ac}}{2a}, \end{array}$$

where

(7) $$\begin{array}{c} a=\left(x_{1}^{2}-\frac{S_{p}^{2}t^{2}}{n_{1}}\right),\\ b=2x_{1}x_{2},\\ c=\left(x_{2}^{2}-\frac{S_{p}^{2}t^{2}}{n_{2}}\right), \end{array}$$

where *x*_1_ and *n*_1_ are the mean and number of measurements obtained by the suggested method, respectively. The same values for the reported method are denoted *x*_2_ and *n*_2_. *S*_*p*_ is the pooled standard deviation and *t* is one-sided *t*-test using a confidence level of 95%. The values of the lower (*θ*_*L*_) and upper (*θ*_*U*_) limits for the determination of NEO in the veterinary drug are demonstrated in Table 3.

### 3.11. Analysis Method Comparison

Compared with other detection methods for neomycin (Table 4), the RRS method has relatively high sensitivity and does not require complicated preprocessing procedures. Moreover, compared with other methods, the RRS method also has the advantages of being cheap, sensitive, and convenient.

## 4. Conclusion

CR interacted with NEO to form an ion association, which greatly improved the RRS strength of the solution. On this basis, the addition of DTAB further improved the strength of RRS. The intensity of RRS had a linear relationship with the concentration of neomycin. And the effect of coexisting ions was covered with BR buffer solution, which made the method have good reproducibility. This method is simple, rapid, and highly sensitive, and can be used to determine the concentration of neomycin in the environment.

## Figures and Tables

**Table 1:** Foreign substance allowances.

Foreign matter	Allowable concentration (*μ*g·mL^−1^)	Relative error (%)	Foreign matter	Allowable concentration (*μ*g·mL^−1^)	Relative error (%)
Glucose	100	−1.3	Starch	80	−2.1
*β*-cyclodextrin	90	−3	Ascorbic acid	50	1.4
L-aspartic acid	100	−2.2	Tryptophan	70	2.3
EDTA	100	2.7	L-serine	90	1.9
L-leucine	80	2.7	L-glutamate	50	−3.4
L-tryptophan	50	−1.5	NH_4_Cl	5	−0.7
KCl	8	1.3	CaCl_2_	10	0.9
ZnSO_4_	10	1.7	CuCl_2_	8	1.4
MnCl_2_	8	3.5	AlCl_3_	10	2.5
FeCl_3_	8	2.4	CdCl_2_	5	−1.4

**Table 2:** Sample testing (*n* = 10).

Sample	Added amount (*μ*g·mL^−1^)	Measured amount (*μ*g·mL^−1^)	Recovery rate ± standard deviation (%)
1	0.2	0.192	96 ± 1.36
2	0.5	0.502	100.4 ± 3.27
3	0.7	0.697	99.5 ± 2.41

**Table 3:** Estimation of NEO in veterinary drugs (QianFang animal protection technology Co., Ltd.) by the proposed spectroscopic method.

Method	% recovery^a^ ± SD	*t*-value^b^	*F*–value^b^	*θ*_*L*_	*θ*_*U*_
RRS	98.80 ± 1.71	0.80	1.99	1.001	1.014
Reported	99.56 ± 1.21				

Note. a: average of five determinations; b: tabulated value at 95% confidence limit; *F* = 6.338; and *t* = 2.306.

**Table 4:** Comparison of the RRS method with other reported detection methods for neomycin.

Method	Linear range	Linear range	Sample pre-treatment	Cost	Reference
LC	0.2∼1.0 mg·kg^−1^	0.10 mg kg^−1^	Centrifugal	Expensive	[6]
LC-MS/MS	0∼1000 *μ*g·kg^−1^	50 *μ*g kg^−1^	PP^a^, LTP^b^ and SPE	Expensive	[7]
HPLC-ELSD	2∼50 mg·L^−1^	0.6 mg L^−1^	LLE	Expensive	[8]
ECL	10^−9^∼10^−5^ M	3.5 × 10^−10^ M	PP^a^	Expensive	[9]
ELISA	1∼1000 *μ*g·L^−1^	2.73 *μ*g L^−1^	Dilution	Expensive	[11]
Fluorescence	2∼1000 g·L^−1^	0.16 g L^−1^	No	Cheap	[12]
RRS	0.07∼1 *μ*g·mL^−1^	0.02 *μ*g mL^−1^	No	Cheap	This article

Note: PP^a^, protein precipitation. LTP^b^, low temperature precipitation.

## Data Availability

Data supporting the findings of this study can be found in the supplementary material of this study.

## Supplementary Materials

Supplementary Materials Figure S1 Effect of adding 1 mL Britton–Robinson buffer solution at different pH values. Congo red: 1.0 × 10^−5^ mol·L^−1^, neomycin: 1.0 *μ*g·mL^−1^, DTAB: 1.0 × 10^−5^ mol·L^−1^ Figure S2 Effect of adding different volumes of pH 6.0 Britton–Robinson buffer solution. Congo red: 1.0 × 10^−5^ mol·L^−1^, neomycin: 1.0 *μ*g·mL^−1^L, DTAB: 1.0 × 10^−5^ mol·L^−1^ Figure S3 Effect of reaction for 30 minutes at different temperatures. 1 mL pH 6.0 Britton–Robinson buffer solution, Congo red: 1.0 × 10^−5^ mol·L^−1^, neomycin: 1.0 *μ*g·mL^−1^, DTAB: 1.0 × 10^−5^ mol·L^−1^ Figure S4 Effect of different concentrations of Congo red. 1 mL pH 6.0 Britton–Robinson buffer solution, neomycin: 1.0 *μ*g·mL^−1^, DTAB 1.0 × 10^−5^ mol·L^−1^ Figure S5 Effect of different concentrations of DTAB. 1 mL pH 6.0 Britton–Robinson buffer solution, Congo red: 1.5 × 10^−5^ mol·L^−1^, neomycin: 1.0 *μ*g·mL^−1^ Figure S6 Stabilization time. 1 mL pH 6.0 Britton–Robinson buffer solution, Congo red: 1.5 × 10^−5^ mol·L^−1^, neomycin: 1.0 *μ*g·mL^−1^, DTAB: 1.0 × 10^−5^ mol·L^−1^ Figure S7 Influence of addition order. 1 mL pH 6.0 Britton–Robinson buffer solution, Congo red: 1.5 × 10^−5^ mol·L^−1^, neomycin: 1.0 *μ*g·mL^−1^, DTAB: 1.0 × 10^−5^ mol·L^−1^ Figure S8 Influence of different ionic strengths. 1 mL pH 6.0 Britton–Robinson buffer solution, Congo red: 1.5 × 10^−5^ mol·L^−1^, neomycin: 1.0 *μ*g·mL^−1^, DTAB: 1.0 × 10^−5^ mol·L^−1^ Table S1 Order of Addition.

## References

[1] Hanko V. P., Rohrer J. S. (2010). Suitability of a liquid chromatography assay of neomycin sulfate to replace the microbiological assay for neomycin in USP monographs. *Journal of Pharmaceutical and Biomedical Analysis*.

[2] Stypulkowska K., Blazewicz A., Fijalek Z., Warowna-Grzeskiewicz M., Srebrzynska K. (2013). Determination of neomycin and related substances in pharmaceutical preparations by reversed-phase high performance liquid chromatography with mass spectrometry and charged aerosol detection. *Journal of Pharmaceutical and Biomedical Analysis*.

[3] Peng J., Tang J., He R., He Y., Xiao Y. (2013). Validation of the high performance liquid chromatography method for the analysis of neomycin sulfate with resonance rayleigh scattering detection. *Analytical Methods*.

[4] Ouyang H., Liang A., Jiang Z. (2018). A simple and selective resonance rayleigh scattering-energy transfer spectral method for determination of trace neomycin sulfate using Cu_2_O particle as probe. *Spectrochimica Acta Part A: Molecular and Biomolecular Spectroscopy*.

[5] Xiao C., Liu J., Yang A. (2015). Colorimetric determination of neomycin using melamine modified gold nanoparticles. *Microchimica Acta*.

[6] Posyniak A., Zmudzki J., Niedzielska J. (2001). Sample preparation for residue determination of gentamicin and neomycin by liquid chromatography. *Journal of Chromatography A*.

[7] Arsand J. B., Jank L, Martins M. T. (2016). Determination of aminoglycoside residues in milk and muscle based on a simple and fast extraction procedure followed by liquid chromatography coupled to tandem mass spectrometry and time of flight mass spectrometry. *Talanta*.

[8] Megoulas N. C., Koupparis M. A. (2004). Enhancement of evaporative light scattering detection in high-performance liquid chromatographic determination of neomycin based on highly volatile mobile phase, high-molecular-mass ion-pairing reagents and controlled peak shape. *Journal of Chromatography A*.

[9] Feng D., Tan X., Wu Y. (2019). Electrochemiluminecence nanogears aptasensor based on MIL-53(Fe)@CdS for multiplexed detection of kanamycin and neomycin. *Biosensors and Bioelectronics*.

[10] Peng J., Wang Y., Liu L., Kuang H., Li A., Xu C. (2016). Multiplex lateral flow immunoassay for five antibiotics detection based on gold nanoparticle aggregations. *RSC Advances*.

[11] Jin Y., Jang J. W., Lee M. H., Han C. H. (2006). Development of ELISA and immunochromatographic assay for the detection of neomycin. *Clinica Chimica Acta*.

[12] Wan Y.-C., Liu Y.-J., Liu C. (2018). Rapid determination of neomycin in biological samples using fluorescent sensor based on quantum dots with doubly selective binding sites—sciencedirect. *Journal of Pharmaceutical and Biomedical Analysis*.

[13] Zhou G., Wang F., Wang H., Kambam S., Chen X. (2013). Colorimetric and fluorometric detection of neomycin based on conjugated polydiacetylene supramolecules. *Macromolecular Rapid Communications*.

[14] Sierra-Rodero M., Fernández-Romero J. M., Gómez-Hens A. (2012). Determination of aminoglycoside antibiotics using an on-chip microfluidic device with chemiluminescence detection. *Microchimica Acta*.

[15] Salem H., Abo Elsoud F. A., Heshmat D., Magdy A. (2021). Resonance Rayleigh scattering technique-using erythrosine B, as novel spectrofluorimetric method for determination of anticancer agent nilotinib: application for capsules and human plasma. *Spectrochimica Acta Part A: Molecular and Biomolecular Spectroscopy*.

[16] Li C., Liu S., Liu Z., Hu X. (2011). Study on the interaction between verapamil hydrochloride and Eosin Y by absorption, fluorescence and resonance rayleigh scattering spectra and their analytical applications. *Journal of Fluorescence*.

[17] Qasem M., El Kurdi R., Patra D. (2020). Glutathione-capped CuO nanoparticles for the determination of cystine using resonance Rayleigh scattering spectroscopy. *Microchimica Acta*.

[18] Liu S. P., Kong L. (2006). Determination of sodium carboxymethyl cellulose by resonance rayleigh scattering method with ethyl violet. *Journal of Southwest China Normal University(Natural Science Edition)*.

[19] Almahri A., Abdel-Lateef A. Q. (2021). Applying different spectroscopic techniques for the selective determination of daclatasvir using merbromin as a probe: applications on pharmaceutical analysis. *Luminescence*.

[20] Abdel-Lateef A. K., Derayea S. M., El-Deen D. A. M. N., Almahri A., Oraby M. (2021). Investigating the interaction of terbinafine with xanthenes dye for its feasible determination applying the resonance rayleigh scattering technique. *Royal Society Open Science*.

[21] Liu Z., Liu S., Wang L., Peng J., He Y. (2009). Resonance Rayleigh scattering and resonance non-linear scattering method for the determination of aminoglycoside antibiotics with water solubility CdS quantum dots as probe. *Spectrochimica Acta Part A: Molecular and Biomolecular Spectroscopy*.

[22] Qiao M., Li C., Shi Y., Liu S., Liu Z., Hu X. (2015). Study on interactions of aminoglycoside antibiotics with calf thymus DNA and determination of calf thymus DNA via the resonance rayleigh scattering technique. *Luminescence the Journal of Biological & Chemical Luminescence*.

[23] Al-Onazi W. A., Abdel-Lateef M. A. (2022). Catalytic oxidation of O-phenylenediamine by silver nanoparticles for resonance rayleigh scattering detection of mercury (II) in water samples. *Spectrochimica Acta Part A: Molecular and Biomolecular Spectroscopy*.

[24] Althabaiti S. A., Aazam E. S., Khan Z., Bashir O. (2016). Aggregation of Congo red with surfactants and Ag-nanoparticles in an aqueous solution. *Spectrochimica Acta Part A: Molecular and Biomolecular Spectroscopy*.

[25] Spólnik P., Stopa B., Piekarska B. (2007). Research article: the use of rigid, fibrillar congo red nanostructures for scaffolding protein assemblies and inducing the formation of amyloid-like arrangement of molecules. *Chemical Biology & Drug Design*.

[26] Rashidi-Alavijeh M., Javadian S., Gharibi H., Moradi M., Tehrani-Bagha A. R., Shahir A. A. (2011). Intermolecular interactions between a dye and cationic surfactants: effects of alkyl chain, head group, and counterion. *Colloids and Surfaces A: Physicochemical and Engineering Aspects*.

[27] Puchtler H., Sweat F., Kuhns J. G. (1964). On the binding of direct cotton dyes by amyloid. *Journal of Histochemistry and Cytochemistry*.

[28] Meloan S. N., Puchtler H. (1978). Demonstration of amyloid with Mesitol WLS-congo red: application of a textile auxiliary to histochemistry. *Histochemistry*.

[29] Gao D., He N., Tian Y., Chen Y., Zhang H., Yu A. (2007). Determination of bovine serum albumin using resonance light scattering technique with sodium dodecylbenzene sulphonate–cetyltrimethylammonium bromide probe. *Spectrochimica Acta Part A: Molecular and Biomolecular Spectroscopy*.

[30] Sun Z., Song M., Zou W., Su Z., Bai Y. (2020). Resonance rayleigh scattering spectra study on the interactions of chito-oligosaccharides with acid blue 119 and their analytical applications—sciencedirect. *Microchemical Journal*.

[31] Zou W., Song M., He J., Qiu P. P., Bai Y. (2021). A resonance rayleigh scattering and fluorescence quenching dual-channel sensor for sensitive detection of chitosan based on eosin Y. *Analytical and Bioanalytical Chemistry*.

[32] Huang C. Z., Li K. A., Tong S. Y. (1996). Determination of nucleic acids by a resonance light-scattering technique with *α*, *β*, *γ*, *δ*-tetrakis[4- (trimethylammoniumyl)phenyl]porphine. *Analytical Chemistry*.

[33] Huang C. Z., Zhu J. X., Li K. A., Tong S. Y. (1997). Determination of albumin and globulin at nanogram levels by a resonance light-scattering technique with *α*, *β*, *γ*, *δ*-Tetrakis(4-sulfophenyl)porphine. *Analytical Sciences*.

[34] Li J., Wang J., Chang H., Wei W. (2017). Resonance rayleigh scattering and resonance nonlinear scattering of the palladium(II)–acetazolamide chelate with eosin Y and their analytical application. *Spectroscopy Letters*.

[35] Zhang W., Ma C., Su Z., Bai Y. (2016). Resonance rayleigh scattering method for highly sensitive detection of chitosan using aniline blue as probe. *Spectrochimica Acta Part A: Molecular and Biomolecular Spectroscopy*.

[36] Abdel-Lateef M. A., Almahri A., Derayea S. M., Samir E. (2020). Xanthene based resonance rayleigh scattering and spectrofluorimetric probes for the determination of cyclobenzaprine: application to content uniformity test. *Journal Reviews in Analytical Chemistry*.

[37] Abdel-Lateef M. A., Almahri A. (2021). Micellar sensitized resonance rayleigh scattering and spectrofluorometric methods based on isoindole formation for determination of Eflornithine in cream and biological samples. *Spectrochimica Acta Part A Molecular and Biomolecular Spectroscopy*.

[38] Almahri A., Abdel‐Lateef M. A., Samir E., Derayea S. M., Hamd M. A. E. (2020). Resonance rayleigh scattering and spectrofluorimetric approaches for the selective determination of rupatadine using erythrosine B as a probe: application to content uniformity test. *Luminescence*.

