# Supplementary material for: Enhancement of Congo Red-Neomycin Resonance Rayleigh Scattering by Dodecyl Trimethyl Ammonium Bromide and its Application
Source: Int J Anal Chem. 2022 Aug 27;2022:6970747. doi: 10.1155/2022/6970747 (PMC9440822; doi:10.1155/2022/6970747)
Supplement: Supplementary Materials — Figure S1 Effect of adding 1 mL Britton–Robinson buffer solution at different pH values. Congo red: 1.0 × 10^−5^ mol·L−1, neomycin: 1.0 μg·mL−1, DTAB: 1.0 × 10^−5^ mol·L−1 Figure S2 Effect of adding different volumes of pH 6.0 Britton–Robinson buffer solution. Congo red: 1.0 × 10^−5^ mol·L−1, neomycin: 1.0 μg·mL−1L, DTAB: 1.0 × 10^−5^ mol·L−1 Figure S3 Effect of reaction for 30 minutes at different temperatures. 1 mL pH 6.0 Britton–Robinson buffer solution, Congo red: 1.0 × 10^−5^ mol·L−1, neomycin: 1.0 μg·mL−1, DTAB: 1.0 × 10^−5^ mol·L−1 Figure S4 Effect of different concentrations of Congo red. 1 mL pH 6.0 Britton–Robinson buffer solution, neomycin: 1.0 μg·mL−1, DTAB 1.0 × 10^−5^ mol·L−1 Figure S5 Effect of different concentrations of DTAB. 1 mL pH 6.0 Britton–Robinson buffer solution, Congo red: 1.5 × 10^−5^ mol·L−1, neomycin: 1.0 μg·mL−1 Figure S6 Stabilization time. 1 mL pH 6.0 Britton–Robinson buffer solution, Congo red: 1.5 × 10^−5^ mol·L−1, neomycin: 1.0 μg·mL−1, DTAB: 1.0 × 10^−5^ mol·L−1 Figure S7 Influence of addition order. 1 mL pH 6.0 Britton–Robinson buffer solution, Congo red: 1.5 × 10^−5^ mol·L−1, neomycin: 1.0 μg·mL−1, DTAB: 1.0 × 10^−5^ mol·L−1 Figure S8 Influence of different ionic strengths. 1 mL pH 6.0 Britton–Robinson buffer solution, Congo red: 1.5 × 10^−5^ mol·L−1, neomycin: 1.0 μg·mL−1, DTAB: 1.0 × 10^−5^ mol·L−1 Table S1 Order of Addition. [file 6970747.f1.docx]

**Supplemental files for**

**Enhancement of Congo Red-Neomycin Resonance Rayleigh Scattering by Dodecyl Trimethyl Ammonium Bromide and Its Application**

Fanfan Zhang^1,2^, Yangyang Chen^1,2^, Dan Zhang^1,2^, Yang Jia^1,2^, Junsheng Meng^3^, Lirong Jiang^3^, Shengke Yang^1,2^*

1. Key Laboratory of Subsurface Hydrology and Ecology in Arid Areas, Ministry of Education, Chang’an University, Xi’an 710054, China.
2. School of Water and Environment, Chang'an University, Xi'an 710054, China.
3. China Jikan Research Institute of Engineering investigations and Design Co, LTD, Xi'an 710000, China.

Correspondence should be addressed to Shengke Yang; [ysk110@126.com](mailto:chzxzdd@126.com%20(D.Z);%20ysk110@126.com).

**Number of pages: 7**

**Number of figures: 8**

**Number of table: 1**

**Contents:**

**Figures:**

**Fig. S1** Effect of adding 1 mL Britton-Robinson buffer solution at different pH values.

**Fig. S2** Effect of adding different volumes of pH 6.0 Britton-Robinson buffer solution.

**Fig. S3** Effect of reaction for 30 minutes at different temperatures.

**Fig. S4** Effect of different concentrations of Congo red.

**Fig. S5** Effect of different concentrations of DTAB.

**Fig. S6** Stabilization time.

**Fig. S7** Influence of addition order.

**Fig. S8** Influence of different ionic strengths.

**Table:**

**Table S1** Order of Addition





Fig. S1 Effect of adding 1 mL Britton-Robinson buffer solution at different pH values. Congo red: 1.0 × 10^-5^ mol·L^-1^，neomycin: 1.0 μg·mL^-1^, DTAB: 1.0 × 10^-5^ mol·L^-1^





Fig. S2 Effect of adding different volumes of pH 6.0 Britton-Robinson buffer solution. Congo red: 1.0 × 10^-5^ mol·L^-1^, neomycin: 1.0 μg·mL^-1^L, DTAB: 1.0 × 10^-5^ mol·L^-1^





Fig. S3 Effect of reaction for 30 minutes at different temperatures. 1 mL pH 6.0 Britton-Robinson buffer solution, Congo red: 1.0 × 10^-5^ mol·L^-1^, neomycin: 1.0 μg·mL^-1^, DTAB: 1.0 × 10^-5^ mol·L^-1^





Fig. S4 Effect of different concentrations of Congo red. 1 mL pH 6.0 Britton-Robinson buffer solution, neomycin: 1.0 μg·mL^-1^, DTAB 1.0 × 10^-5^ mol·L^-1^





Fig. S5 Effect of different concentrations of DTAB. 1 mL pH 6.0 Britton-Robinson buffer solution, Congo red: 1.5 × 10^-5^ mol·L^-1^, neomycin: 1.0 μg·mL^-1^





Fig. S6 Stabilization time. 1 mL pH 6.0 Britton-Robinson buffer solution, Congo red: 1.5 × 10^-5^ mol·L^-1^, neomycin: 1.0 μg·mL^-1^, DTAB: 1.0 × 10^-5^ mol·L^-1^





Fig. S7 Influence of addition order. 1 mL pH 6.0 Britton-Robinson buffer solution, Congo red: 1.5 × 10^-5^ mol·L^-1^, neomycin: 1.0 μg·mL^-1^, DTAB: 1.0 × 10^-5^ mol·L^-1^





Fig. S8 Influence of different ionic strengths. 1 mL pH 6.0 Britton-Robinson buffer solution, Congo red: 1.5 × 10^-5^ mol·L^-1^, neomycin: 1.0 μg·mL^-1^, DTAB: 1.0 × 10^-5^ mol·L^-1^

Table S1 Order of Addition

| No. | addition sequence |
| --- | --- |
| 1 | Congo red - Neomycin - DTAB |
| 2 | Congo red - DTAB - Neomycin |
| 3 | Neomycin - Congo red - DTAB |
| 4 | Neomycin - DTAB - Congo red |
| 5 | DTAB - Congo red - Neomycin |
| 6 | DTAB - Neomycin - Congo red |
